# Assessment of Patient Knowledge and Potential Screening Gaps in Vitamin B12 Deficiency

**DOI:** 10.7759/cureus.87830

**Published:** 2025-07-13

**Authors:** Alec J Lippmann, Muhammad Awan, Parth Patel, Shayan Samavati, Benjamin E Lippmann, Martin Clemmons

**Affiliations:** 1 Clinical Sciences, Alabama College of Osteopathic Medicine, Dothan, USA; 2 Research, Alabama College of Osteopathic Medicine, Dothan, USA; 3 Medicine, Alabama College of Osteopathic Medicine, Dothan, USA; 4 Psychiatry, Private Practice, Lakeland, USA; 5 Internal Medicine, Alabama College of Osteopathic Medicine, Dothan, USA

**Keywords:** autoimmune disease, b12 testing, questionnaire, vitamin b12, vitamin b12 deficiency

## Abstract

Introduction

Vitamin B12 is an essential nutrient that functions as a coenzyme in the nervous system and is critical for the synthesis of myelin and neurotransmitters. Vitamin B12 deficiency disrupts these processes and is associated with significant neurological impairment. Our study aimed to evaluate patient awareness of vitamin B12 deficiency and perceived health risks, as well as to identify potential gaps in its screening.

Methods

The study was conducted using an anonymous 11-item questionnaire distributed to clinics throughout Alabama and Florida, as well as circulated on various social media platforms. The survey analyzed participants' symptoms, risk factors, knowledge, and perceived importance of B12 deficiency.

Results

Less than a quarter of symptomatic respondents had undergone B12 testing in the past year, with nearly all those tested having a recognized risk factor and only one individual being tested solely based on symptoms. A vast majority of participants reported at least one risk factor for B12 deficiency, with participants with autoimmune conditions reporting nearly twice the number of symptoms as others. Despite moderate concern about B12 deficiency, over half of our participants self-reported low confidence in their understanding of the condition.

Conclusion

Our findings highlight the gap between patient symptoms and diagnostic action, as well as a deficit in patient awareness and understanding of B12 deficiency. Symptom-centered screening strategies and improved public awareness are warranted to improve early detection and management of vitamin B12 deficiency.

## Introduction

Vitamin B12 is an essential nutrient that serves as a coenzyme in the nervous system and is involved in the synthesis of myelin and neurotransmitters. B12 also plays a vital role in the synthesis of deoxyribonucleic acid (DNA) and ribonucleic acid (RNA), as well as in the conversion of methylmalonyl CoA to succinyl CoA. A deficiency of vitamin B12 can disrupt these fundamental processes, and B12 deficiency has been associated with profound detrimental effects on the function and well-being of patients [1].

There are four stages of vitamin B12 deficiency. The first stage is serum depletion, where the amount of B12 bound to delivery protein transcobalamin II (TCII) is low. The second stage is cell depletion, characterized by decreasing holohaptocorrin levels and low red blood cell vitamin B12 concentrations. During these stages, serum B12 levels appear normal, and the only way to accurately measure vitamin B12 deficiency is by examining levels of holotranscobalamin II (holoTCII). During stage 3, DNA synthesis is slowed due to the unavailability of free vitamin B12, which is required for the regeneration of tetrahydrofolate (THF) and succinyl-CoA, resulting in elevated serum homocysteine and methylmalonate concentrations, respectively [2]. Stage 4 of vitamin B12 deficiency presents as macrocytic anemia in the patient. HoloTCII falls below its expected range within days after intestinal absorption of vitamin B12 stops, compared to total serum vitamin B12, which falls below its normal limits during stages 3 and 4 of the disease [2].

Peripheral neuropathy is a specific pathology associated with vitamin B12 deficiency. The neuronal demyelination that occurs during vitamin B12 deficiency-associated neuropathy is thought to be caused by decreased levels of S-adenosylmethionine (SAM) in the body, the synthesis of which relies upon vitamin B12 [1]. Vitamin B12 deficiency has also been associated with a range of neuropsychiatric problems, including depression, mania, delirium, and cognitive impairment [3]. In one study done by Sayadnasiri et al., they found vitamin B12 deficiency to be an overlooked risk factor for falling in elderly patients with chronic psychiatric disorders [4]. The prevalence of B12 deficiency in the United States varies by age, ranging from at least 3% of individuals between 20 and 39 years of age being affected to at least 6% of people aged 60 or older. Marginal depletion of vitamin B12 is seen in a greater proportion of the American population, affecting more than 20% of individuals 60 years or older [5].

Vitamin B12 deficiency has been shown to increase in elderly subjects because, as humans age, the stomach produces less hydrochloric acid. Without adequate amounts of hydrochloric acid, the human body may only be able to absorb 1-5% of vitamin B12 via passive diffusion [5]. Vegetarians and vegans are also populations known to be vulnerable to B12 deficiency due to their limited or absent consumption of B12-rich foods (e.g., beef, salmon, and eggs) [6]. Patients with autoimmune disorders are another group associated with an increased risk of B12 deficiency because of autoimmune gastritis and decreased production of intrinsic factors (i.e., pernicious anemia) [7]. Individuals who have undergone gastric surgery are also at an elevated risk of B12 deficiency due to multiple factors, including reduced gastric acid secretion and alterations in gastrointestinal anatomy [8].

Some medications commonly taken by patients have been shown to have detrimental effects on vitamin B12 levels after prolonged use, like proton pump inhibitors (PPIs), metformin, and bile acid sequestrants. One proposed mechanism by which metformin interferes with vitamin B12 absorption is by inhibiting the calcium ion (Ca2+), which is required for intrinsic factor-mediated vitamin B12 absorption in the ileum. PPIs block the gastric hydrogen-potassium adenosine triphosphatase (H+K+ ATPase) enzyme, thereby decreasing gastric acid secretion. The lack of gastric acid is thought to decrease the release of vitamin B12 from the proteins in food, leading to decreased absorption availability in the ileum [9]. Bile acid sequestrants have also been shown to cause vitamin B12 depletion because bile is necessary for the renal and hepatic uptake of vitamin B12 from the intestinal lumen [10].

Vitamin B12 deficiency is commonly diagnosed through a peripheral blood smear, which demonstrates macrocytic anemia characterized by hypersegmented polymorphs and an increased mean corpuscular volume. A significant issue in the diagnosis of B12 deficiency is that most patients are commonly tested for vitamin B12 upon diagnosis of macrocytic anemia, either through a complete blood count or a peripheral blood smear, rather than being tested based on neurological disorders such as neuropathy. Therefore, many B12-deficient patients are left undiagnosed with a higher risk of developing more severe neurological disorders [5]. Moreover, healthcare providers may not consider a diagnosis of vitamin B12 deficiency when treating neuropathy. They may not even check B12 levels due to the difficulty and lack of accuracy associated with testing for B12 deficiency without the gold standard [5,11].

This study aims to evaluate patient awareness of vitamin B12 deficiency and identify potential gaps in its screening and diagnosis. Through a brief questionnaire, we seek to assess patients' understanding of B12 deficiency, identify any associated risk factors and perceived symptoms among participants, and measure the level of concern patients associate with B12 deficiency as a health issue. Ultimately, our goal is to encourage more proactive screening for B12 deficiency, especially in individuals with one or more risk factors, such as gastric surgery and advanced age. Furthermore, this study underscores the broader need for the development of a reliable, gold-standard diagnostic test to enhance the detection and management of this condition.

## Materials and methods

This was a cross-sectional, observational study conducted over a three-month period from February to April 2025. The study was carried out in collaboration with primary care practices located in Alabama and Florida. Ethical approval was obtained from the Alabama College of Osteopathic Medicine (ACOM) Institutional Review Board, which classified the study as Exempt under Category 2 for survey-based research. Electronic informed consent was provided by all participants before beginning the survey.

Recruitment was performed using a multimodal approach. Flyers containing a quick-response (QR) code and direct link to the survey, along with a short description of the study, were distributed by clinic staff and displayed in areas frequently visited by patients, such as waiting rooms and examination rooms. Additionally, the survey was shared on LinkedIn, Reddit, and Facebook using a standardized post explaining the study’s purpose and eligibility criteria.

Prior to the full launch, the questionnaire underwent pilot testing with a small group (n = 10) of non-medical individuals to assess clarity and ease of use. Based on their feedback, revisions were made to improve readability and flow. Content validity was confirmed by two physicians and a medical school faculty member with experience in internal medicine and nutritional disorders. Internal consistency for scaled items (e.g., confidence in knowledge) was acceptable (Cronbach’s alpha = 0.78).

The questionnaire, distributed via the Qualtrics software (Qualtrics, Provo, UT), included 11 items assessing symptoms, risk factors, perceived severity, and confidence in understanding of vitamin B12 deficiency. Responses were anonymized, and basic demographic data (age and sex assigned at birth) were collected. Information about the privacy and confidentiality of the questionnaire was presented at the beginning of the Qualtrics survey.

Although this was an exploratory study, a post hoc sample size calculation was performed. Based on the observed effect size (Cohen’s d = 1.302) between groups with and without autoimmune conditions, a minimum of 20 participants per group would be required to achieve 80% power at a significance level of 0.005. The final sample size (n = 41) yielded a calculated power of 82.7%, indicating sufficient statistical power despite the modest sample size.

The survey would close at 100 responses or after three months, whichever came first. The study population consisted of individuals who met the inclusion criteria of having internet access, being 19 years or older, and having experienced any of the specific symptoms of B12 deficiency listed in the survey within the past year. These criteria were selected to concentrate on at-risk patients to the greatest extent and to reduce potential recall bias. The questionnaire was made available in February 2025 for participants to complete. A total of 59 responses were received by the end of the three-month study period, and the Qualtrics survey was subsequently closed. All survey data were filtered and analyzed using Microsoft Excel (Microsoft Corporation, Redmond, WA) and GraphPad (https://www.graphpad.com/; GraphPad Software, San Diego, CA) programs.

Screening questions were then provided to determine if respondents met the inclusion criteria to continue the questionnaire. If the participant did not meet the inclusion criteria mentioned above, the survey would automatically close. The screening questions would also be used for data analysis. Initially, the questionnaire provided three possible age groups for participants to choose from: 0-18, 19-59, and 60 and above. Participants who selected the 0-18 age group were automatically excluded from the questionnaire.

On the other hand, individuals 19 and older were further questioned on possible symptoms of B12 deficiency experienced in the past year. Multiple symptoms could be selected by the participants, including muscle weakness, fatigue, paresthesia, difficulty with ambulation, and lack of energy, among others. If the participants denied experiencing any symptoms, they would be excluded from the survey.

Following the screening questions, respondents were requested to provide their gender, with the option to decline to answer. Participants were then asked if they had any of the following risk factors for B12 deficiency: autoimmune disease (e.g., Hashimoto's disease), use of a proton pump inhibitor for three or more years, a history of gastric bypass surgery, or a strict vegetarian or vegan diet for three or more years. Participants were also asked if they had received a vitamin B12 deficiency test in the past year (e.g., vitamin B12 levels and methylmalonic acid levels).

Participants were then requested to self-report their confidence in understanding vitamin B12 deficiency on a scale from 1 to 5, where 1 indicated "very confident" and 5 signified "very unconfident." Finally, respondents were asked to rate the seriousness of a health concern related to vitamin B12 deficiency on a scale of 1 to 5, with 1 indicating "no concern at all" and 5 indicating "a major concern."

Data were analyzed using Microsoft Excel and GraphPad Prism. Descriptive statistics were used to summarize categorical variables (frequencies, percentages) and continuous variables (mean, standard deviation). The primary comparison of symptom counts between participants with and without autoimmune conditions was performed using an unpaired two-tailed t-test. A p-value less than 0.005 was considered statistically significant. Confidence intervals (95%) were reported to indicate the precision of estimates. Post hoc power analysis was also conducted to evaluate the adequacy of the sample size based on the observed effect size.

## Results

Survey response rate

A total of 59 responses were received during the survey period. The survey data were filtered to remove any responses that were incomplete or completed in under 60 seconds. Responses from participants who were under 19 years of age or did not select any of the specific symptoms of B12 deficiency were also filtered out. After filtering the data, a total of 41 responses remained (68.3%).

Participant demographics

Participants were requested to self-report their gender and age to understand patient demographics better. Of the participants, 73.2% (n = 30) were female, 19.5% (n = 8) of our respondents were male, 4.9% (n = 2) identified as non-binary/third gender, and 2.4% (n = 1) preferred not to disclose. Of the participants, 73.2% (n = 30) were aged between 19 and 59, and 26.8% (n = 11) of our respondents were 60 years or older (Table 1).

**Table 1:** Demographics of the participants.

Variables	Category	Frequency	Percent
Gender	Female	30	73.2
	Male	8	19.5
	Non-binary/third gender	2	4.9
	Prefer not to say	1	2.4
Age	19–59	30	73.2
	60+	11	26.8

Symptoms experienced by the survey population

Respondents were asked to self-report any symptoms of B12 deficiency they had experienced in the past year. Because this question was multi-answer and patients could list more than one symptom, the total number of responses (n = 141) was greater than the number of participants (n = 41).

**Figure 1:**
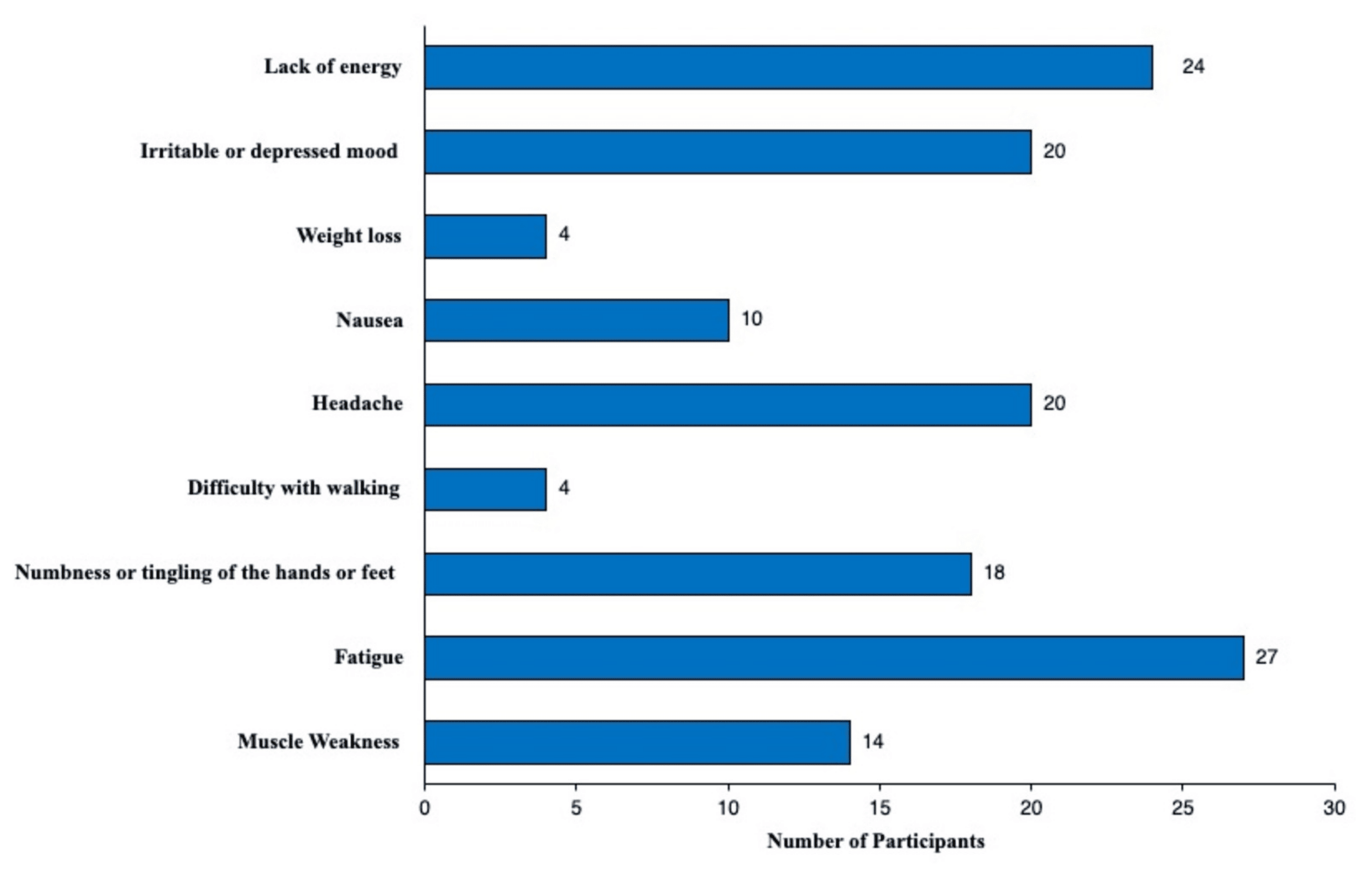
Patient-reported symptoms of B12 deficiency. Self-reported symptoms of B12 deficiency from patients with or without risk factors for a B12 deficiency. Patients who denied symptoms were excluded from the study, and this figure.

Fatigue, reported by 65.9% (n = 27) of participants, and lack of energy, reported by 58.5% (n = 24) of participants, were the most frequently reported symptoms. More severe side effects, including weight loss and difficulty walking, were reported by four participants each (Figure 1).

The rate of participants with symptoms who have been tested for B12 deficiency

**Figure 2:**
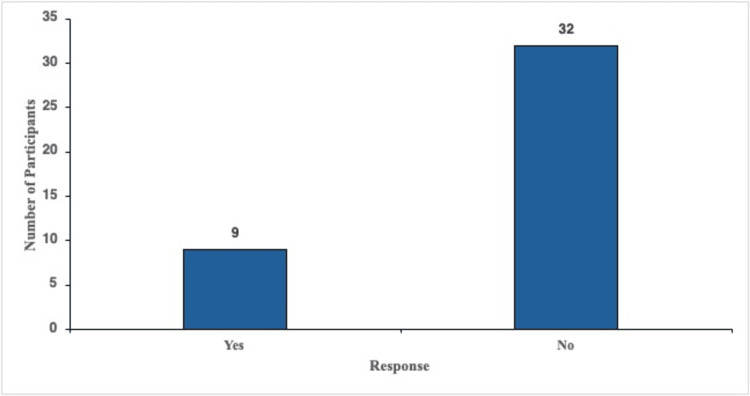
Patients reporting whether they were tested for vitamin B12 deficiency in the past year. Responses of patients when asked if they had received a vitamin B12 deficiency test (e.g., methylmalonic acid levels, serum vitamin B12 levels, and homocysteine levels) in the past year. Only patients who experienced symptoms of B12 deficiency were included.

Participants with symptoms were asked to self-report whether they had been tested for vitamin B12 deficiency in the past year via methylmalonic acid, serum vitamin B12 levels, or homocysteine levels test. This was asked via a yes-or-no question; the specific method of testing was not asked for. Of the participants, 21.9% (n = 9) had been tested for vitamin B12 deficiency in the past year. Of the nine participants tested, 88.9% (n = 8) had risk factors for vitamin B12 deficiency, indicating that only one participant was tested purely based on symptoms (Figure 2).

The rate of participants with symptoms and risk factors versus those with symptoms and without risk factors

**Figure 3:**
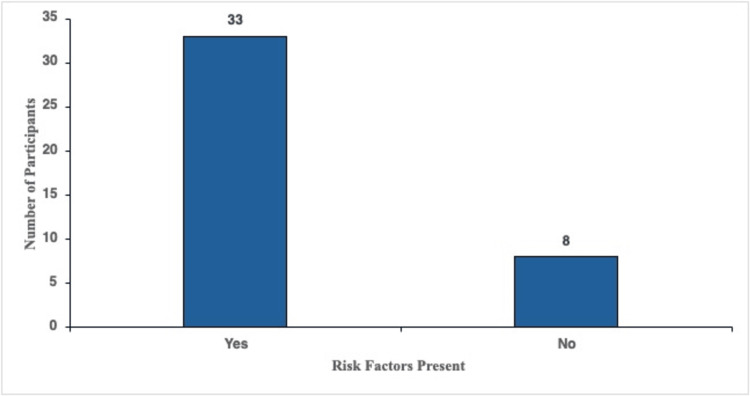
Symptomatic patients who had risk factors for vitamin B12 deficiency versus those who had no risk factors.

Participants were asked about risk factors at the beginning of the survey. These risk factors included being diagnosed with an autoimmune condition, prolonged PPI use, having undergone gastric surgery, and following a strict vegetarian or vegan diet for a prolonged period. Participants were also asked about their age, and although not directly posed as a risk factor, being 60 or above is considered a risk factor; therefore, this was also counted as a risk factor. Of the participants, 80.5% (n = 33) had risk factors, and 19.5% (n = 8) had no risk factors at all (Figure 3).

Relationship between the number of symptoms and autoimmune condition

**Figure 4:**
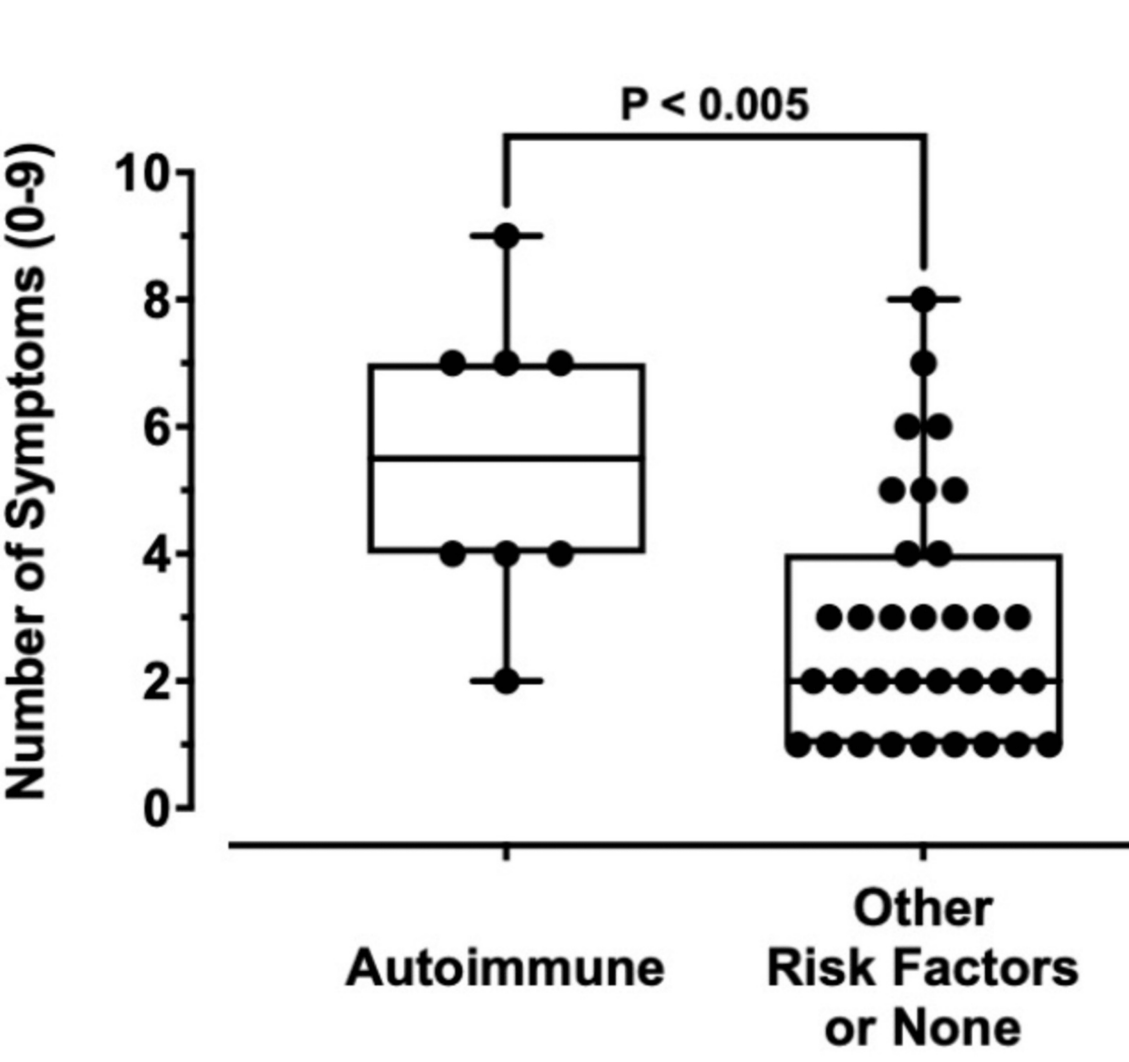
Boxplots demonstrating the number of symptoms experienced by those with autoimmune conditions as compared to those without. The average number of symptoms experienced by those with autoimmune conditions was 5.5 (n = 8, range = 0-9) and 2.91 (n = 33, range = 0-9) for those without an autoimmune condition, representing a difference of 2.59 ± 0.78 (p-value < 0.005). The ends of the upper and lower whiskers indicate the maximum and minimum values of each dataset, respectively. The open boxes represent the interquartile ranges (IQR), and the horizontal lines indicate the medians of the datasets.

Highlighting autoimmune conditions such as pernicious anemia is essential, as they represent not only a leading cause of B12 deficiency but are also the most severe form, yet patients are often unaware of their risk or the need for lifelong, non-oral treatment. This underscores a key gap in patient understanding that may delay diagnosis and appropriate care. To highlight this, the number of symptoms experienced by those with an autoimmune condition (Figure 4, left) was compared to the number of symptoms experienced by those without an autoimmune condition (Figure 4, right). The number of possible symptoms could range from zero to nine, with nine indicating a more severe vitamin B12 deficiency. The most reported number of symptoms for those without an autoimmune condition was one (n = nine), with 27.3% of individuals in that category experiencing only one symptom. The most frequently reported number of symptoms in the autoimmune group was bimodal, with four (n = three) and seven (n = three) being the most reported numbers of symptoms. Each represented 37.5% of participants in the autoimmune category.

The average number of symptoms experienced by individuals with an autoimmune condition was 5.50, and the average number of symptoms experienced by those without an autoimmune condition was 2.91, resulting in a total difference of 2.59 ± 0.78 (95% CI: -4.18 to -1.00; p-value = 0.0021) (Figure 4).

Importance of vitamin B12 deficiency to the survey population

**Figure 5:**
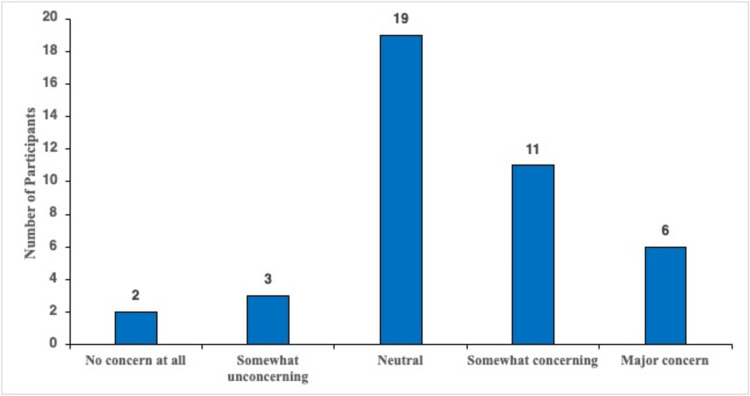
Participants rating their level of concern regarding B12 deficiency.

Participants were asked to self-report how much of a health concern they feel that vitamin B12 deficiency is. The five possible responses are depicted in Figure 5. Of the participants, 14.6% (n = six) felt vitamin B12 deficiency was a "major concern," and 26.8% (n = 11) felt that the deficiency was "somewhat concerning" as a health issue. A total of 46.3% (n = 19) felt "neutral" about vitamin B12 deficiency as a health concern, 7.3% (n = three) believed it to be "somewhat concerning," and 4.9% (n = two) felt B12 deficiency was "no concern at all" (Figure 5).

Knowledge of vitamin B12 deficiency in the survey population

**Figure 6:**
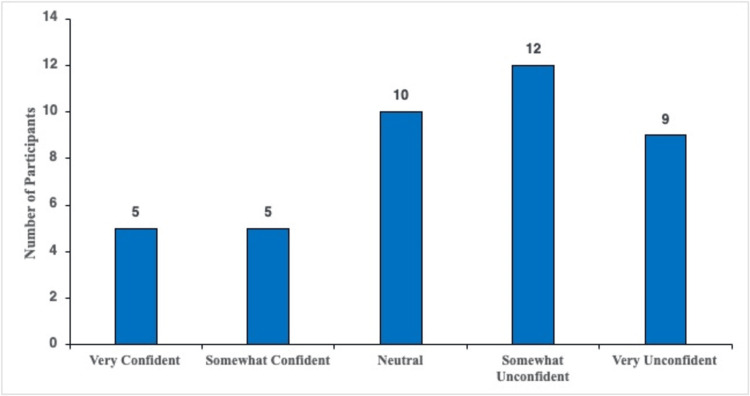
Participants' perceived levels of confidence about their understanding of vitamin B12 deficiency.

Participants were asked to self-report their level of confidence in understanding vitamin B12 deficiency. The five possible response options are depicted in Figure 6. Of the participants, 12.2% (n = five) answered that they felt "very confident" about their understanding of vitamin B12 deficiency, and 12.2% (n = five) answered "somewhat confident." A total of 24.4% (n = 10) answered that they felt "neutral" about their understanding of vitamin B12 deficiency, 29.3% (n = 12) stated that they felt "somewhat unconfident" about their vitamin B12 deficiency understanding, and 21.9% (n = nine) answered "very unconfident" (Figure 6).

## Discussion

In our study, we analyzed patient understanding and perception of vitamin B12 deficiency through a questionnaire study. Data were gathered on subjective symptoms of vitamin B12 deficiency experienced, what risk factors patients had that could predispose them to vitamin B12 deficiency, and whether patients had been tested for vitamin B12 deficiency. One of the most significant findings from our survey is that fatigue (65.9%) and lack of energy (58.5%) were the most reported symptoms potentially associated with vitamin B12 deficiency. Although nonspecific, these are the hallmark signs of early deficiency and tend to align with clinical presentations that are often attributed to lifestyle or stress-related factors. Severe manifestations, such as weight loss and difficulty walking, which may reflect advanced neurological involvement, were reported by only four participants. This pattern indicated that severe B12 deficiency is relatively uncommon in our sample or often underrecognized by our participants.

In addition to fatigue and low energy, which were the most frequently reported symptoms in our cohort, vitamin B12 deficiency can present with a much broader spectrum of manifestations. These include cognitive impairment, memory difficulties, irritability, depression, glossitis, paresthesia, lightheadedness, visual disturbances, and even infertility in rare cases [12,13]. Neurological symptoms, especially paresthesia and impaired proprioception, can occur even in the absence of anemia, and may not fully reverse even after B12 repletion if diagnosis is delayed [12]. This underscores the high stakes of early detection: once demyelination of the spinal cord (subacute combined degeneration) sets in, patients may be left with permanent disability, including persistent numbness, balance disorders, or cognitive decline. In our study, the relatively low reporting of such advanced symptoms may reflect underrecognition, but it also serves as a reminder of how insidious B12 deficiency can be, presenting subtly yet progressing silently toward irreversible harm.

According to the National Institutes of Health (NIH) Office of Dietary Supplements, symptoms of vitamin B12 deficiency typically begin with fatigue, weakness, and loss of appetite. They may progress to neurologic symptoms such as numbness, balance issues, and cognitive changes if left untreated [12]. Since early symptoms were more common than severe features, and participants lacked confidence in their understanding, these results suggest the need for improved education to diagnose and treat vitamin B12 deficiency before it worsens.

Our survey revealed a potential blind spot in everyday practice. Only 21.9% of symptomatic respondents had been evaluated for vitamin B12 deficiency in the previous 12 months, and 88.9% of the ordered tests were conducted on individuals who also carried a classic risk factor (age of at least 60 years, long-term use of metformin or PPIs, vegan diet, gastric surgery, etc.). Put differently, only one participant, approximately 2% of the entire symptomatic sample, was tested based solely on symptoms (Figure 2).

That pattern is not unique to our cohort. In a Veterans Affairs study of 3,687 adults on long-term metformin, only 37% ever received a serum B12 check despite well-established links between metformin and malabsorption [13]. A larger multicenter analysis (13,489 metformin users) found a similarly modest 44.9% testing rate and a median delay of almost three years before the first assay was drawn [14]. Even when clinicians are polled directly, fewer than half report that they "routinely" screen symptomatic patients for B12 deficiency [15].

Current guidance may have a role in the infrequency of B12 testing. The 2024 NICE guideline on vitamin B12 deficiency recommends investigation when "typical symptoms are present and a recognized risk factor is documented," implicitly suggesting that symptoms alone are insufficient to trigger investigation [16]. Our data and those from the metformin and physician-practice studies suggest that such risk-based algorithms leave a sizable group of symptomatic, ostensibly low-risk adults untested and potentially untreated.

Taken together, these findings strengthen the case for a more symptom-centered approach in primary care. Simple decision-support prompts (e.g., ordering serum B12 with or without methylmalonic acid when a patient reports persistent fatigue or paresthesia) and automatic alerts for pharmacies dispensing high-risk medications could help close the gap, ensuring that biochemical deficiency is picked up before hematologic or neurologic damage becomes irreversible.

In our survey, 80.5% of symptomatic adults reported at least one recognized trigger for vitamin B12 deficiency, including long-term PPI therapy, an autoimmune condition, previous gastric surgery, a strict vegetarian or vegan diet, or age of at least 60 years (Figure 3). Only 19.5% of the participants appeared to be risk-free based on the questionnaire. That skew is hardly a surprise. Population studies show that vitamin B12 stores tend to decline steadily after the sixth decade [17]. This is particularly relevant given the current US demographics, where older adults already make up nearly one in five Americans (approximately 18% were at least 65 years old in 2023, projected to reach 22% by 2040) [18]. Importantly, PPIs sit among the 20 most-prescribed drugs in the United States, with roughly 113 million prescriptions a year [19], further amplifying the public health relevance of B12 deficiency.

However, what may be more interesting is the small group who were risk-free on our questionnaire (Figure 3). A closer examination suggests that some of the eight respondents who appeared "risk-free" may not have been risk-free at all. Instead, they may have been exposed to factors not captured by our questionnaire. For instance, long-term metformin use is a well-established cause of malabsorption, with recent systematic reviews confirming significantly higher odds of biochemical B12 depletion in metformin-treated diabetics [12]. Recreational nitrous oxide (N₂O) use can induce a functional B12 deficiency by oxidizing the cobalt center of the cobalamin molecule. This disrupts its biological activity even when serum levels appear normal. A case series published in 2024 highlights acute myeloneuropathy in otherwise healthy young adults using N₂O recreationally [20]. Furthermore, several gastrointestinal conditions are known to interfere with B12 absorption. Celiac disease and Crohn's disease reduce absorption in the small intestine; small-intestinal bacterial overgrowth allows microbes to consume the vitamin first, and chronic pancreatitis deprives B12 of the pancreatic enzymes needed to release it for uptake [12,21,22].

Hence, it can be reasonably deduced that some of the eight respondents who appeared "risk-free" may have carried these underlying risk factors, and our findings likely underestimate the actual burden of risk factors in symptomatic adults. Expanding future surveys to include medication histories, subtle malabsorption states, or lifestyle exposures could reveal that the "no-risk" category is still smaller. A broader checklist or even an open-ended prompt would help clinicians avoid missing less obvious but clinically relevant factors that contribute to vitamin B12 deficiency.

When examining the number of symptoms experienced by individuals with an autoimmune condition and comparing it to those without one, the average number of symptoms was nearly twice as high, at 5.5, compared to 2.91 for those without an autoimmune condition, reflecting a difference of 2.59 (Figure 4). Pernicious anemia, an autoimmune condition that impairs vitamin B12 absorption, is the most common cause of clinically overt B12 deficiency [23]. This deficiency manifests in fatigue, neuropathy, mood and cognitive disturbances, gastrointestinal issues, and other systemic symptoms, many of which overlap significantly with the broader symptom profile of autoimmune disorders [23]. Pernicious anemia has a higher incidence of other autoimmune diseases in both individuals with pernicious anemia and their family members [24]. This may help explain why participants in our study with autoimmune disorders reported a greater number of symptoms compared to those with other risk factors for vitamin B12 deficiency, as pernicious anemia is associated with more severe and clinically impactful B12 deficiency than other etiologies [5].

As evident from Figure 5, our results highlight the hesitancy among survey participants regarding vitamin B12 deficiency as a health concern. Our data showed that approximately 46.3% reported feeling "neutral" about the health issue, while 12.2% felt it was "somewhat unconcerning" or "not a concern at all." On the other hand, a combined 41.4% viewed it as either "somewhat concerning" or a "major concern." These findings suggest that, despite uncertainty, a significant proportion of respondents view vitamin B12 deficiency as a concern, demonstrating an awareness of its significance.

Importantly, this perceived concern stands in contrast to participants' low confidence in their understanding of the condition (Figure 6). A total of 24.4% felt either "very" or "somewhat" confident in their knowledge of vitamin B12 deficiency, while 51.2% felt "somewhat unconfident" or "very confident." The gap between concern and confidence highlights a disconnect between awareness and understanding the significance of vitamin B12 deficiency.

Studies have shown that, despite an estimated 10-20% prevalence of subclinical vitamin B12 deficiency in older adults, public awareness remains limited [25]. As a result, this survey highlights the need for increased public awareness, not only to inform people about B12 deficiency but also to provide knowledge on both early and late symptoms of vitamin B12 deficiency. Educational campaigns aimed at older adults, long-term medication users, and individuals with restrictive diets could increase awareness and prompt self-advocacy for testing. In clinical practice, routine symptom-driven screening, even in the absence of traditional risk factors, should be considered, especially when patients report fatigue, paresthesia, or neurologic symptoms.

Limitations

The sample size, potential for recall bias, and confounding variables are limitations of our study that must be addressed. The small sample size (n = 41) may be attributed to the three-month study period and the recruitment of primary care physicians in Florida and Alabama. Small sample sizes can introduce a higher risk of errors, lower power, and lower statistical significance [26]. In our study, the null hypothesis was rejected because our p-value (0.0021) was under the alpha value of 0.005, indicating that our study had sufficient power despite the small sample size. This may be explained by the magnitude of the association (standardized effect size = -1.302) rather than the inherent power of our study (82.7% by post hoc power analysis). Since the effect of having an autoimmune disease on the severity of vitamin B12 deficiency is strong, it can be detected even with a smaller sample size like ours. It is also important to acknowledge that while we rejected the null hypothesis, there is always a possibility of a type 1 error [26]. Our study's alpha value was set to 0.005, indicating a very low likelihood of type 1 error. Even so, the sample size, and thereby the statistical power and significance, may be improved in our future questionnaires by lengthening the study period to at least a year and expanding the survey to primary care clinics outside of Florida and Alabama, allowing it to be distributed to a broader range of patients. Social media use for recruitment should also be limited, as online survey respondents may be younger, resulting in fewer participants over 60 being captured by our study.

Although we attempted to minimize the risk of recall bias by inquiring about the symptoms experienced by participants in the past year, the bias nonetheless remains a significant issue. With any study that requests patients to recall information, inaccurate memories of symptoms experienced may significantly alter results. Furthermore, considering how vitamin B12 deficiency can alter mental status, patient perceptions of their symptoms may be affected. In our further studies, recall bias can be reduced by restricting the query to a shorter time frame (e.g., having experienced symptoms within the last six months instead of the last 12 months).

One potential confounding variable in our study is that the symptoms used to assess vitamin B12 deficiency, such as fatigue or neurological complaints, are nonspecific and may also result from unrelated health conditions. This overlap may have affected the accuracy of symptom attribution and limited the specificity of our findings. This can be improved in future studies by screening for common comorbid conditions that can cause similar symptoms, such as depression, anxiety, and neurological conditions, and statistically adjusting for or excluding respondents. We also did not assess for medication use beyond PPI exposure, leaving open the possibility that other medications, such as selective serotonin reuptake inhibitors (SSRIs), antipsychotics, or antihypertensives, may have contributed to symptoms like fatigue, confusion, or dizziness. Including a more detailed medication history could help account for these potential confounders.

Lastly, all symptom and risk factor data were self-reported, which may introduce both measurement bias and misclassification bias. Without confirmation via medical records or laboratory data, the accuracy of reported symptoms and testing history cannot be guaranteed. Future studies could improve data reliability by incorporating objective clinical or laboratory validation of self-reported information.

## Conclusions

The results of our study demonstrate a gap between patient-reported symptoms and diagnostic action, highlighting potential deficits in current screening guidelines for B12 deficiency. Although participants reported symptoms consistent with B12 deficiency, many of these patients remained untested for the condition. Furthermore, public understanding of B12 deficiency was notably low despite the high prevalence of the condition. These findings underscore the importance of symptom-centered screening strategies and improved public awareness to promote early detection. This is especially critical given that untreated B12 deficiency can result in irreversible neurologic complications. Delayed diagnosis not only increases the risk of permanent disability but may also contribute to higher healthcare utilization due to preventable diagnostic workups and long-term care needs. Therefore, timely recognition and treatment of B12 deficiency are essential to prevent avoidable morbidity and reduce associated healthcare costs. Clinicians should consider incorporating routine vitamin B12 screening in symptomatic patients, even without obvious risk factors, as early detection may prevent irreversible complications. Future guideline revisions may benefit from a more symptom-inclusive diagnostic algorithm, especially in primary care. Public health efforts should also focus on reducing knowledge gaps and ensuring high-risk populations are both aware of and screened for B12 deficiency. Further research is warranted to build on our findings and support the development of more inclusive and proactive screening measures.

## Figures and Tables

**Figure 7:**
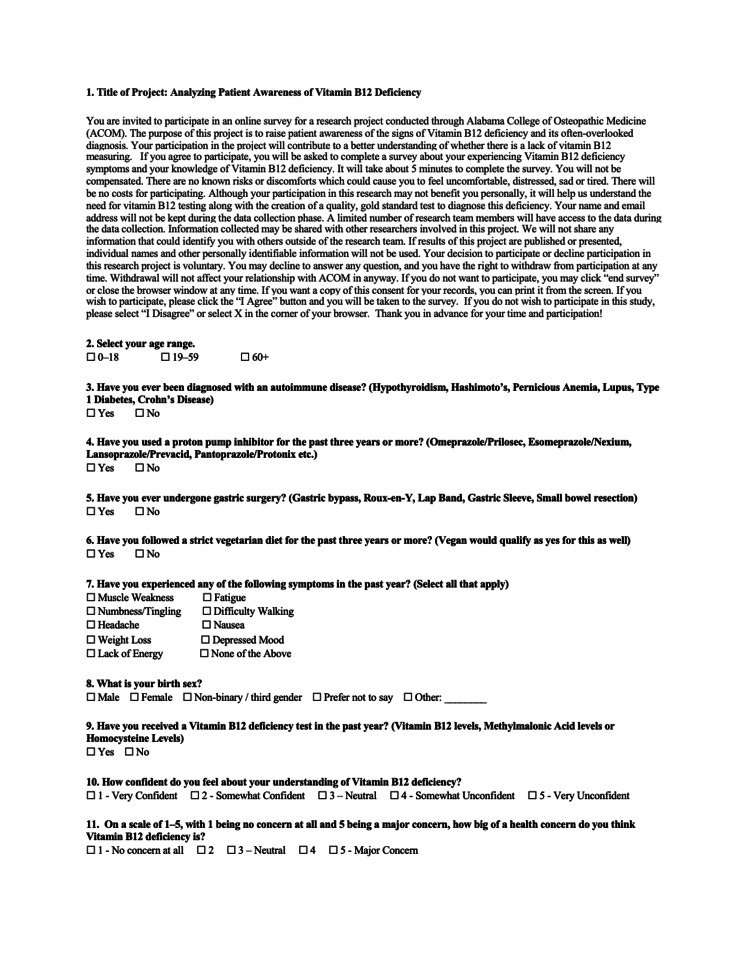
Copy of the online questionnaire administered to participants. Questionnaire data were collected through the Qualtrics survey software (Qualtrics, Provo, UT).
